# Automated array-CGH optimized for archival formalin-fixed, paraffin-embedded tumor material

**DOI:** 10.1186/1471-2407-7-43

**Published:** 2007-03-07

**Authors:** Simon A Joosse, Erik H van Beers, Petra M Nederlof

**Affiliations:** 1 Division of Experimental Therapy, the Netherlands Cancer Institute, Amsterdam, The Netherlands; 2 Department of Pathology, the Netherlands Cancer Institute, Amsterdam, The Netherlands

## Abstract

**Background:**

Array Comparative Genomic Hybridization (aCGH) is a rapidly evolving technology that still lacks complete standardization. Yet, it is of great importance to obtain robust and reproducible data to enable meaningful multiple hybridization comparisons. Special difficulties arise when aCGH is performed on archival formalin-fixed, paraffin-embedded (FFPE) tissue due to its variable DNA quality. Recently, we have developed an effective DNA quality test that predicts suitability of archival samples for BAC aCGH.

**Methods:**

In this report, we first used DNA from a cancer cell-line (SKBR3) to optimize the aCGH protocol for automated hybridization, and subsequently optimized and validated the procedure for FFPE breast cancer samples. We aimed for highest throughput, accuracy, and reproducibility applicable to FFPE samples, which can also be important in future diagnostic use.

**Results:**

Our protocol of automated array-CGH on archival FFPE ULS-labeled DNA showed very similar results compared with published data and our previous manual hybridization method.

**Conclusion:**

This report combines automated aCGH on unamplified archival FFPE DNA using non-enzymatic ULS labeling, and describes an optimized protocol for this combination resulting in improved quality and reproducibility.

## Background

Array CGH has become a successful and valuable tool for the analysis of chromosome copy-number alterations including the detection of sub-megabase alterations and has been applied to *e.g*. cell-lines, (tumor) tissues, and lymphocytes [1-5]. The power of aCGH technology to detect low-level copy number changes is critically dependent on DNA quality (*e.g*. DNA fragmentation and cross-links) and sample heterogeneity. Therefore, selection of DNA of sufficient quality, especially when using FFPE material, is of great importance for aCGH [6]. Furthermore, whole genome amplification may be performed when insufficient DNA is available from a sample [7-9]. In addition to sample quality, enzymatic labeling protocols decrease average DNA size further which results in increased noise due to non-specific binding [10], especially when the average PCR length of the sample template drops below 200 bp [6]. As an alternative, chemical labeling protocols with cyanin cis-platinum-labeled DNA resulted in good aCGH results [11], also for FFPE archival samples [6]. One of the challenges of aCGH is its lower hybridization signal-intensity compared with metaphase-CGH. Based on literature and our previous experiments we hypothesized that the hybridization improves with increasing effective concentration of labeled DNA, which is limited by the viscosity of the hybridization mixture, as well as by the duration and temperature of the hybridization. In this study we performed in total 70 aCGH hybridizations across these parameters and report how these impact on the CGH profile quality. We first used SKBR3 DNA to explore hybridization variables that are important for aCGH and then show how this expertise can be applied to FFPE primary human tumors.

There have been earlier reports on array CGH of FFPE material [6-8,12-14] and other reports on automated hybridization [15]. This report however, is the first that combines automated hybridization of FFPE tumor material on a BAC array, using non-enzymatic labeling and provides a method without formamide in the post-hybridization washes.

## Methods

DNA was isolated from the breast cancer cell-line SKBR3 (obtained from ATCC) or from FFPE tumor tissue with at least 70% tumor cells as described before [6]. Two micrograms of total genomic DNA were labeled with ULS-Cy5 according to the manufacturers' instructions (Kreatech Biotechnology, Amsterdam). Reference DNA was isolated from lymphocytes of six apparently healthy women, pooled, and sonicated as was done with the SKBR3 genomic DNA to obtain fragments of similar size distribution as DNA from FFPE material (approximately 300–800 bp). Two micrograms of pooled reference DNA were labeled with ULS-Cy3. Corning CodeLink^® ^slides containing the human 3.5 k BAC/PAC genomic clone set in triplicate were used as before [6]. As optimization target, we used CGH profiles of 6 FFPE tumors containing at least 70% tumor cells and the SKBR3 cell-line profile, obtained by the manual hybridization method described before [6]. Automated hybridizations were done in 63.5 × 21 mm chambers in a Tecan HS4800 Pro™ hybridization station, which uses liquid agitation during hybridization. Experiments involving human tissues were conducted with permission of our institutes' medical ethical advisory board.

### Optimal (pre-) hybridization mixture

Labeled sample and reference DNA were pooled with 125 μg C_0_t-1 DNA (Roche, 1581–074) and precipitated. The pellet was dissolved in 140 μl 0.22 μm filtered hybridization buffer (50% formamide, 15% dextran sulphate (USB 14489, Mw 40–50 kDa), 0.1% Tween20, 2 × SSC, 10 mM Tris pH 7.4, and 25 mM EDTA) and 10 μl (100 μg/μl) yeast tRNA (Sigma, R-8759). The pre-hybridization solution consisted of 400 μg single stranded sheared herring sperm DNA (Sigma, D7290) and 125 μg C_0_t-1 DNA dissolved in 150 μl hybridization buffer. Both hybridization and pre-hybridization mixtures were dissolved at 37°C continuously shaking at 650 rpm (Eppendorf Thermomixer) for at least one hour, denatured for 10 min at 95°C and spun for 1 min at 14000 rpm (Eppendorf centrifuge) to pellet potential particles prior to injecting 120 μl pre-hybridization mixture followed by 120 μl sample mixture into the hybridization chamber.

### Optimal automated hybridization

Optimal hybridization parameters for the hybridization station: **step 1**; wet the array with 2 × SSC for 30s at 37°C, no soak. **Step 2**; 120 μl pre-hybridization solution was slowly injected and incubated for 1 hour at 37°C, agitation set at 'high'. **Step 3**; 15s wash at 37°C with 2 × SSC, no soak. **Step 4**; 120 μl sample mixture was injected and hybridized for 72 hours at 37°C, agitation set at 'high'. **Step 5**; 12 × (1 min wash, 1 min soak) with 2 × SSC + 0.1% SDS at 37°C. **Step 6**; 6 × (1 min wash + 1 min soak) with 2 × SSC + 0.1% SDS at 68°C. **Step 7**; 2 × (1.5 min wash + 1 min soak) with 2 × SSC at 68°C. **Step 8**; 1,5 min wash with 0.1 × SSC at 23°C, no soak. **Step 9**; 2 min with nitrogen gas at 23°C. Slides were scanned with an Agilent DNA Microarray Scanner BA on the same day. Data processing included signal intensity measurement in ImaGene Software followed by median pintip (*c.q*. subarray) normalization and plotting in custom Matlab code as before [6].

### Data analysis

Three statistics were used to determine the quality of the hybridization, the CGH profile, and to compare experiments with each other. For each CGH profile, we calculated the variance across all log2 ratios relative to the ratios of the underlying true ploidy levels as estimated by CGH-segmentation [16], secondly, we defined the dynamic range as the difference between the minimum log2 ratio and the maximum log2 ratio calculated by CGH-segmentation [16], and the average of all the standard deviations of the triplicate spot measurements of each probe was used as a third statistic. Thus, an optimal CGH profile has a low variance to give a better estimate of the copy number level, a high dynamic range to give the best resolution of copy numbers and a low average standard deviation for reproducibility.

### GEO

Microarray data have been deposited in NCBIs Gene Expression Omnibus and are accessible through GEO Series accession number GSE7122.

## Results and Discussion

### Optimization of aCGH on a hybridization station

Further automation of CGH is indispensable to meet the demand for higher quality, higher throughput, and improved reproducibility. We here describe automated and reproducible array-CGH on FFPE material. As optimization goals we aimed to reproduce results from manual hybridizations and published results, to minimize the variance, to maximize dynamic range, to minimize standard deviation of the triplicate spot measurements, and to maximize signal-to-noise.

It is difficult to determine the quality of aCGH profiles without an independent methodology to verify gains and losses. Therefore, we chose to use the widely studied cell-line SKBR3 as a model for which chromosomal aberrations have been well documented [2,3,17], although the existence of minor sub-clone related alterations cannot be ruled out. In a previous study [6], we performed manual hybridizations of over one hundred BAC arrays that helped us to develop the quality criteria that were now used to optimize automated hybridization. In this study we describe multiple hybridizations that were performed in synchronous pairs with one variable tested in each run, including hybridization duration of 24, 48, or 72 hours, hybridization temperature of 37, 42 or 45°C, pre- and post-hybridization wash temperatures of 37, 42, 45, 46, 65 or 68°C, viscosity of the hybridization mixture with 7, 10, 15, 17.5, or 20% dextran sulphate of 5, 10, or 50 kDa average molecular weight, pH 6, 7 or 8 of the hybridization mixture and with or without pre-hybridization. All hybridization parameters studied are relevant to nucleic acid hybridization in general, and here optimized for the 3.5 k BAC arrayCGH platform and may thus be different for other platforms. Hybridizations were done with genomic DNA isolated from the well-described SKBR3 cells or from FFPE breast tumor archival sections to optimize and validate the protocol.

### Optimal conditions for automated hybridization

Using 2 μg unamplified sample DNA from FFPE tissue and 2 μg reference DNA both CyDye labeled, incubation duration was optimal at 72 hours at 37°C after pre-hybridization with a mixture of herring sperm and C_0_t-1 DNA for 1 hour at 37°C. The optimal hybridization buffer contained 15% 50 kDa dextran sulphate. Washing was performed as described in Material and Methods. The steps that led to this protocol are described in detail below.

### Optimization model SKBR3 cell-line

**Figure 1:**
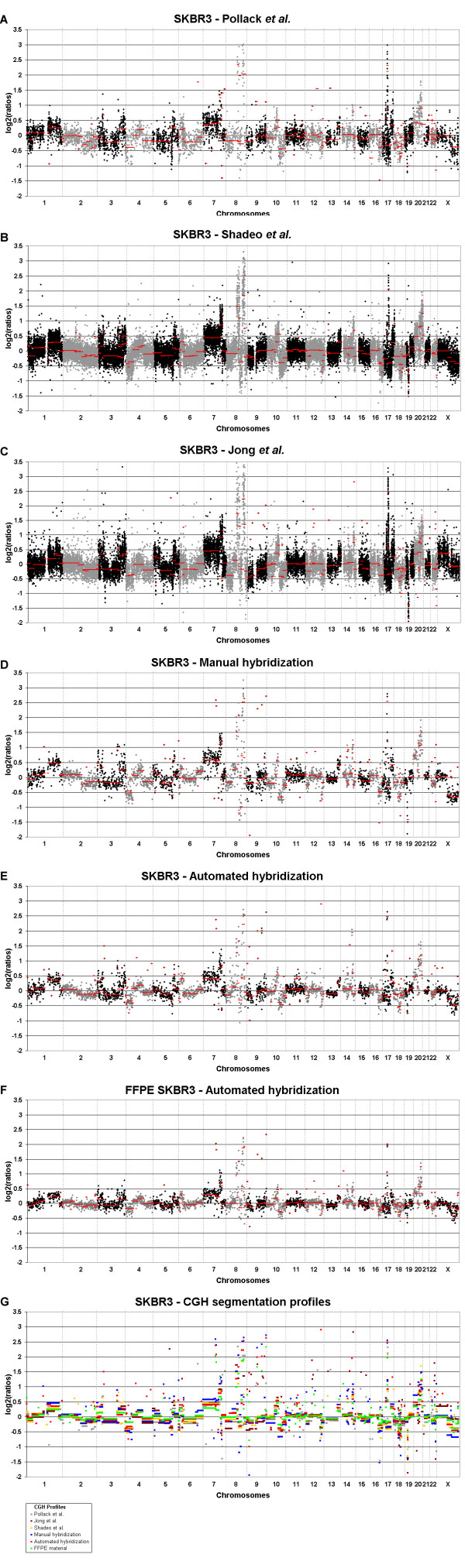
**SKBR3 CGH profiles obtained by various methods**. Chromosomes 1 to X (*X-axis*, alternate shading per chromosome) versus the log2 ratios (*Y-axis*) for the breast cancer cell-line SKBR3, hybridized by Pollack *et al*. (PNAS 1999) on a 6.7 k cDNA micro array, redrawn form values available on-line (**A**), hybridized by Shadeo and Lam (Breast Cancer Res 2006) on a whole-genome tiling path BAC array containing 32,433 overlapping BAC-derived DNA segments (**B**), hybridized by Jong *et al*. (Oncogene 2006) to a human oligonucleotide array containing 28,830 unique genes (**C**), manually hybridized to a 3.5 k BAC/PAC array (**D**), and hybridized using our optimal automated method (**E**). Formalin-fixed paraffin-embedded SKBR3 cell-line hybridized using our automated method (**F**). Red lines in panel A-F represent the breakpoint locations and copy number chances calculated by CGH-segmentation (BMC Bioinformatics 2005). Panel **G **contains all segmentation calls of the profiles depicted in panel A-F.

We used SKBR3 as a model cell-line and compared its CGH profile with published [2,3,17] and our own manual hybridizations. Figure 1A represents the SKBR3 CGH profile published by Pollack *et al*., hybridized to a human cDNA micro array containing 6,691 different mapped human genes [2]. Figure 1B represents the SKBR3 CGH profile published by Shadeo and Lam., hybridized to a whole-genome tiling path BAC array containing 32,433 overlapping BAC-derived DNA segments [3]. Figure 1C represents the SKBR3 CGH profile published by Jong *et al*., hybridized to a human oligonucleotide array containing 28,830 unique genes [17]. Figure 1D represents our manually hybridized SKBR3 CGH profile. Depicted in figure 1E is the very similar SKBR3 CGH profile hybridized with our optimal protocol for the hybridization station except for the slightly different variance and dynamic range. Figure 1F depicts the CGH profile of paraffin embedded SKBR3 (discussed later). To compare these data from different platforms and different methods we looked at the breakpoint locations and copy number estimates as is illustrated in figure 1G. This figure summarizes all the breakpoints and estimated copy number levels as plotted in red in figure 1A–1F calculated by CGH-segmentation [16]. Breakpoint locations and calling of copy number levels (gain, unchanged, heterozygous loss, and homozygous loss) are provided as additional file 1 and 2. Although a lower density 3.5 k BAC array was used, figure 1G illustrates that nearly all aberrations and breakpoint in our results (figure 1D and 1E) are similar to the three published data sets [2,3,17]. We concluded that the dynamic range of both our manual and automated hybridization protocols are adequate to detect single copy number losses and gains. Reproducibility of this automated protocol is shown by replicate hybridizations with a Pearson correlation of 0.85, dynamic ranges of 3.7 versus 3.9, the variances for both experiments 0.11, and the mean standard deviations of 0.07 versus 0.04 (table 1).

### Hybridization duration and temperature

**Figure 2:**
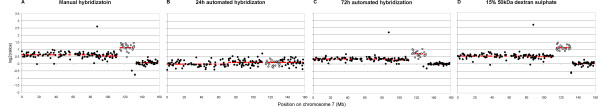
**CGH profiles of manual and three automated methods**. Chromosome 7 (*X-axis*), versus the log2 ratios (*Y-axis*) for the breast cancer cell-line SKBR3 (alternate shading for aberration legibility); manual hybridization (**A**, detail from figure 1D); 24 hours automated hybridization (**B**); 72 hours automated hybridization (**C**); 72 hours automated hybridization using 15% 50kDa Dextran Sulphate (**D**). With increasing hybridization duration from 24 to 72 hours, the CGH profile was more similar to the manual hybridization CGH profile. The aberration in panel C is detected in contrast to panel B (grey), even better with an increased concentration of dextran sulphate from 7 to 15% (**panel D**). Red lines in all panel represents the segmentation calls as calculated by CGH-segmentation.

The effects of hybridization duration and temperature were measured in two experiments using SKBR3 DNA and reference DNA hybridized for 24 or 48 hour at 37°C. Hybridization mixture containing 7% 50 kDa dextran sulphate was used. Figure 2A shows the CGH profile for SKBR3 chromosome 7 (detail from figure 1B, chosen for its clear and multiple aberrations), hybridized according to our manual method. Figure 2B shows the CGH profile after 24 hours of automated hybridization at 37°C. After 24 hours, no aberrations were detected in this CGH profile. After 48 hours only large copy number changes were found and the small deletions and amplifications were not (data not shown). Also, the dynamic range was small (log2 ratios from -1.0 to 2.2) compared with our manual method (from -1.0 to 2.7). CGH profiles after 24 and 48 hours were inferior to our manual method, this is likely due to lower specific signals. To improve this, the hybridization duration was increased to 72 hours and performed at 37, 42 or 45°C. At all three temperatures, the CGH profiles were approaching the quality of the manual hybridization. Figure 2C shows the result of 37°C, as can be seen it is quite similar to the manual hybridization (figure 2A). However, the variances and standard deviations of the triplicate spot measurements increased with hybridization temperature. Mean standard deviations were 0.03, 0.05 and 0.11 at 37, 42 and 45°C respectively. Although 45°C seemed to provide the highest dynamic-range (from -1.1 to 2.6), it was accompanied by the highest noise levels after 72 h (p < 0.00001). The variances were 0.07, 0.11 and 0.13 for 37, 42 and 45°C respectively (table 2). 45°C was therefore excluded from further testing and 37 and 42°C were used to optimize dynamic range in the following experiments.

### Hybridization buffer composition

A major further improvement of the hybridization was obtained by increasing the 50 kDa dextran sulphate concentration from 7 to 15%. Here we describe our results for 10 and 15%. Four hybridizations were done at 37 and 42°C each with 10 or 15% dextran sulphate. The resulting profiles were very similar to each other and to our manual hybridized aCGH. A slight systematic difference was detected in the variance, standard deviation of the triplicates and dynamic range. Hybridizing at 37°C, standard deviations were 0.06 and 0.07 at 10 and 15% dextran sulphate respectively and at both concentrations the variances were 0.11. At 42°C, both the variances increased to 0.14 and 0.12 and the mean standard deviations to 0.10 and 0.09 at 10 and 15% dextran sulphate respectively (table 3). Of these four hybridizations, the best profile is shown in figure 2D, this is at 37°C using 15% dextran sulphate. In this experiment, the dynamic range was 3.7 (from -1.0 to 2.7). We chose not to hybridize at 42°C anymore because of the significant higher variance and standard deviation as a result (p < 0.00001). We chose to use 15% dextran sulphate in further experiments at 37°C because of its low variance and standard deviation and its higher dynamic range compared to using 10% dextran sulphate.

Increasing the concentration of 50 kDa dextran sulphate from 15% to 17.5 or 20% did not further improve the array results. At these concentrations the variance was 0.11 and 0.09 at 17.5% and 20% dextran sulphate respectively, notably at 20% dextran sulphate the dynamic range decreased below 3.5. Elevated concentrations of dextran sulphate render the hybridization mixture viscosity beyond the mixing capability of the hybridization station. This prompted us to evaluate the effect of lower molecular weight dextran sulphate (*i.e*. lower viscosity at the same concentration). We used 5 kDa (Sigma) and 10 kDa (pK Chemicals, Denmark) dextran sulphate at 15, 17.5 or 20% for SKBR3 profiling. All six hybridizations showed inferior dynamic ranges compared with the 50 kDa dextran sulphate experiments, shown in table 4. Therefore, 50 kDa dextran sulphate at a concentration of 15% was used in all subsequent hybridizations.

### Post-hybridization washes

Most wash protocols use large amounts of formamide to wash off non-specifically bound probe. Formamide is a toxic that we wished to exclude from all washes. The wash procedure now consists of: **step 5**; 12 × (1 min wash, 1 min soak) with 2 × SSC + 0.1% SDS at the hybridization temperature of 37, 42 or 45°C (previously discussed), **step 6**; 6 × (1 min wash, 1 min soak) with 2 × SSC + 0.1% SDS at 37, 46 or 65°C, **step 7**; 2 × (1.5 min wash, 1 min soak) with 2 × SSC at 37, 46 or 65°C, **step 8**; 15 sec wash with 0.1 × SSC at 23°C, **step 9**; dry slides for 2 minutes with nitrogen gas at 23°C.

As described before, hybridization was performed at 37, 42 or 45°C. Step 5 was done at these temperatures and results are discussed above. Step 6 and 7 were done at 37 or 46°C, both resulting in inferior profiles compared with the manual hybridization. A large proportion of the deletions and amplifications in the CGH profile could not be detected and the data were essentially as in figure 2B. Increasing the temperatures of steps 6 and 7 to 65°C resulted in good CGH profiles. We concluded that 37°C is the optimum temperature for step 5 and 65°C for steps 6 and 7 when hybridizing a cell-line.

### FFPE tumor tissue optimization and validation

To develop aCGH also as a diagnostic tool, it will be essential to validate its applicability on patient tumor samples and especially on archival FFPE tissue [18]. Extracted DNA from this material is often heavily cross-linked, heterogeneous (*i.e*. mix of cells of different genomic composition), fragmented, and rarely composed of 100% tumor cells. Therefore, aCGH profiles of FFPE material generally have larger variances (defined as the spread around the common levels between adjacent chromosome breakpoints), lower intensities and lower dynamic range compared with hybridizations of cell-line DNA.

To validate our automated hybridization method for FFPE material we compared CGH profiles from unfixed and formalin fixed paraffin embedded SKBR3 cells. Figure 1E shows the CGH profile of the formalin fixed SKBR cell-line. The fresh and FFPE SKBR3 CGH profiles were highly similar and showed a Pearson correlation of 0.87 (table 1). Variances were 0.11 and 0.12, dynamic ranges 3.9 and 3.2, and mean standard deviations 0.04 and 0.05, for fresh and fixed DNA respectively. However, the DNA quality from a paraffin embedded cell-line does not necessarily represent the quality of DNA from archival tumor tissue that can be more than 25 years old and fixed under widely varying conditions.

Therefore, we validated our method on archival material. The first hybridization was done with tumor #1 DNA with or without pre-hybridization after the first wash step (**step 1**: wetting or chamber filling), for 1 hour at 37°C. The pre-hybridization mixture consisted of 400 μg single stranded sheared herring sperm DNA and 125 μg C_0_t-1 DNA dissolved in 150 μl hybridization buffer. With pre-hybridization, signal intensities were almost 50% higher and the mean standard deviation of the triplicate spots 15% lower compared to the protocol without pre-hybridization resulting in good CGH profiles of FFPE material (data not shown). Although CGH profiles of SKBR3 did not improve upon adding pre-hybridization, it clearly benefited CGH profiles of DNA extracted from FFPE patient tissue (data not shown).

**Figure 3:**
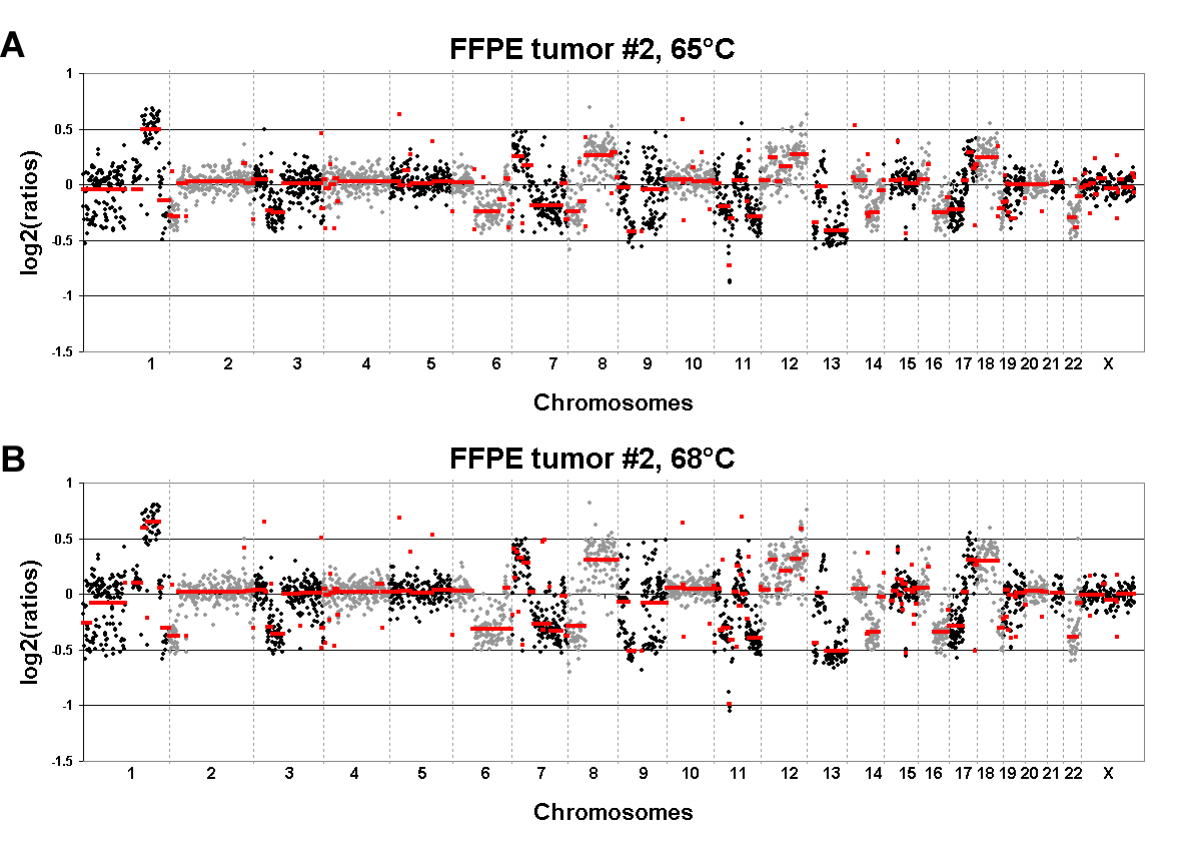
**CGH profiles of one FFPE tumor with post hybridization wash at different temperatures**. CGH profiles with post-hybridization wash steps 6 and 7 at 65°C (**A**) or at 68°C (**B**) of averaged duplicates of one FFPE primary breast tumor, hybridized according to our optimized protocol for automated array-CGH. Chromosomes (*X-axis*, alternate shading per chromosome) versus the log2 ratios (*Y-axis*). At 68°C, dynamic range increased and standard deviation of the triplicate spot measurements decreased compared with 65°C, therefore 68°C was used in our optimal protocol. As can be seen in panel B, the dynamic range and the signal-to-noise are adequate to detect and to distinguish homozygous and heterozygous loss (chromosome 11p), one single-copy number gain (*e.g*. chromosome 7p), multiple-copy numbers gain (chromosome 1q), and unchanged chromosome copy numbers (*e.g*. chromosome 10). Red lines in both panel represents the segmentation calls as calculated by CGH-segmentation.

Because Tris is the only buffering component in the hybridization mixture, we wished to test the possibility that the formamide could react with oxygen and may influence the buffer's pH during storage. To test the effect of pH on the hybridization, six hybridizations with FFPE tumor #2 DNA were done, using hybridization buffers of pH 6, 7 and 8, as measured in the final hybridization buffer. At every pH the CGH profile was very similar and highly reproducible. As can be seen in table 5, standard deviations of the triplicate spot measurements in all six hybridizations were very similar. The variances are lowest at pH 6 but not very different from the variances at pH 7 and 8. For this particular tumor, the maximal CGH-segmentation [16] value was used as dynamic range ("Max CGHseg", table 5), because the homozygous loss on chromosome 11 would have a disproportional contribution to its value (same tumor as in figure 3). These were very similar between experiments. Pearson correlation between the duplicates shows high correlations for all experiments. Therefore, we conclude that aCGH is not very sensitive to pH of the buffer between pH 6 and 8.

Subsequent experiments compared post-hybridization washing at 65°C or 68°C (step 6 and 7) both in duplicate on FFPE extracted material tumor #2. Figure 3A depicts the average profile of two hybridizations washed at 65°C, panel B shows the average CGH profile washed at 68°C, at both temperatures the CGH profiles are very similar. A very small difference could be detected in the mean standard deviation of the triplicate spot measurements as can be seen in table 6, it slightly decreased from 0.04 and 0.05 at 65°C to both 0.03 at 68°C (p < 0.00001). Also the dynamic range kept at similar levels (again the highest ratio calculated by CGH-segmentation [16] was used because of the homologous loss in this tumor in chromosome 11 as depicted in figure 3). Although the benefits of changing the temperature from 65°C to 68°C were small, we decided to wash at 68°C.

**Figure 4:**
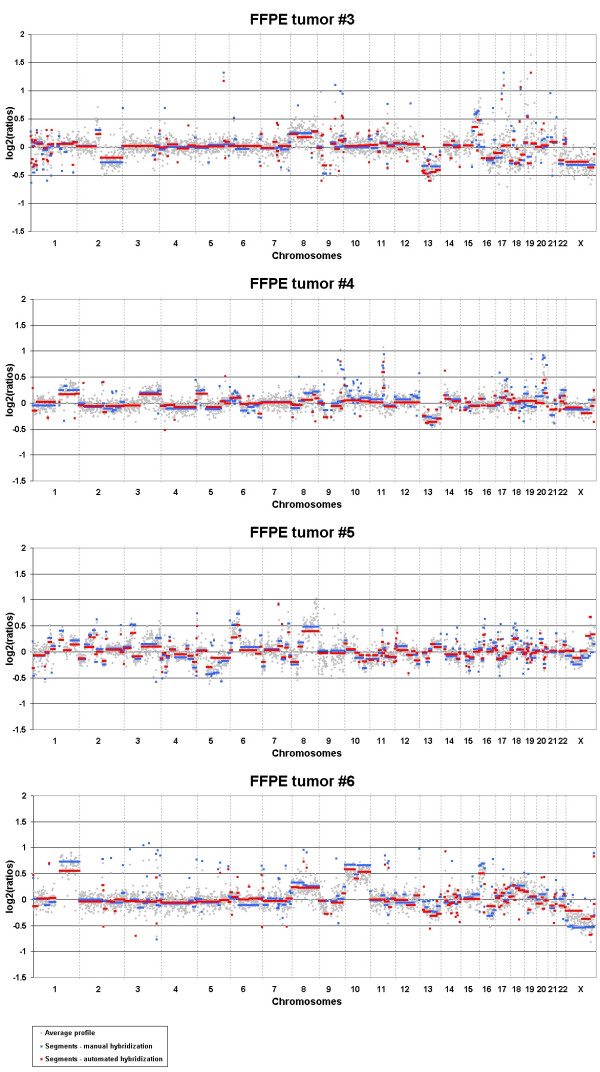
**CGH profiles of four FFPE tumors, hybridized manually and automated**. CGH profiles of four FFPE tumors hybridized using our manual and automated methods. Chromosomes (*X-axis*) versus the log2 ratios (*Y-axis*) averaged over the two methods. Breakpoint locations and copy number estimates calculated by CGH-segmentation of the manual hybridized tumors (blue) and of the automated hybridized tumors (red), and the average CGH profiles (log2 ratios) of the manual and the automated hybridized tumors (grey).

To validate the optimal automated hybridization described above, we hybridized four FFPE samples (tumor #3, 4, 5 and 6) that were previously hybridized using our manual method. Figure 4 shows the CGH profiles of the FFPE tumors (averaged log2 ratios of the manual and the automated hybridization), with very similar breakpoint locations and copy number estimates [16] for each hybridization method. Variance and standard deviation of the triplicate spot measurements improved slightly but significantly (p < 0.00001) for automated compared with manual hybridizations. The dynamic ranges between pairs of manual and automated hybridizations differed by 5%, 7%, 15%, and 0% respectively, and Pearson correlations were 0.82, 0.72, 0.85, and 0.84 (table 7). Although the dynamic ranges are slightly larger due to higher log2 ratios at high-level amplifications using the manual hybridization method (figure 4), these results show that automated and manual CGH profiles are quite similar.

So far, we performed over one hundred automated array-CGH experiments, the oldest archival material used was fixed and embedded in 1971, all with reproducible and high quality results. Figure 3B shows the average profile of one archival FFPE tumor hybridized in duplicate, performed with our optimal protocol for automated aCGH. As can be seen in figure 3, the dynamic range of the hybridizations was adequate to detect and distinguish homozygous and heterozygous loss (chromosome 11p), one single-copy number gain (*e.g*. chromosome 7p), more then one copy number gain (chromosome 1q) and unchanged chromosome copy numbers (*e.g*. chromosome 10) in FFPE tumor tissue.

## Conclusion

To develop an automated hybridization method, we first used the breast cancer cell-line SKBR3 as a model-genome and subsequently optimized and validated the protocol for FFPE breast tumors. Reproducible hybridization results for FFPE tumor tissue were obtained using ULS-labeled unamplified tumor DNA with pre-hybridization, hybridized on a hybridization station at 37°C for 72 hours with a hybridization mixture containing 15% 50 kDa dextran sulphate and post-hybridization washing steps without using formamide. Pre-hybridization did not have a detectable effect on the CGH profile of the cell-line SKBR3 but did improve CGH profiles of FFPE tissue samples. All hybridization parameters studied are optimized for the 3.5 k BAC array-CGH platform but may be different for other platforms. This protocol of automated array-CGH on archival FFPE ULS-labeled DNA outperformed all our manual methods with respect to accuracy, reproducibility, easy of handling, and speed.

## Abbreviations

aCGH = array-Comparative Genomic Hybridization; BAC = Bacterial Artificial Chromosome; bp = base pairs; FFPE = Formalin-Fixed, Paraffin-Embedded; ULS = Universal Linkage System

## Competing interests

The author(s) declare that they have no competing interests.

## Authors' contributions

SJ performed all experiments, data analyses, participated in the study design and wrote the manuscript. EvB helped in writing and data analyses, and participated in the study design. PN participated in the study design and coordination and helped to draft the manuscript. All authors read and approved the final manuscript.

## Pre-publication history

The pre-publication history for this paper can be accessed here:



## Supplementary Material

Additional file 1 This file includes the aberrations calculated by CGH-segmentation for the SKBR3 CGH profiles hybridized by Pollack *et al*., Shadeo and Lam, Jong *et al*., manually, automatically, and hybridized FFPE SKBR3. Calling copy number changes by setting thresholds allowed comparison of different hybridization platforms and methods. Click here for file

Additional file 2 This picture shows the copy number calls (*Y-axis*) from SKBR3 CGH profiles per chromosome (*X-axis*), hybridized by Pollack *et al*., Shadeo and Lam, Jong *et al*., manually, automatically, and hybridized FFPE SKBR3. Gain at 1, unchanged at 0, heterozygous loss at -1, and homozygous loss at -2. Click here for file

## Figures and Tables

**Table 1:** Manual and automated hybridization of SKBR3

**Sample**	**Hyb method**	**Variance**	**StDev**	**DR**	**Correlation**
SKBR3	manual	0.13	0.05	3.7	

SKBR3	automated	0.11	0.04	3.9	0.85
SKBR3	automated	0.11	0.07	3.7	

FFPE SKBR3	automated	0.12	0.05	3.2	0.87

Variance (**Variance**), standard deviation (**StDev**), dynamic range (**DR**) and Pearson correlation (**Correlation**) of SKBR3 performed using our manual and our automated hybridization method (**Hyb method**).

**Table 2:** Hybridization duration and temperature

**Sample**	**Duration**	**Temp.**	**Variance**	**StDev**	**DR**
SKBR3	24	37	0.13	0.11	1.9
SKBR3	48	37	0.12	0.06	3.1
SKBR3	72	37	0.07	0.03	3.1
SKBR3	72	42	0.11	0.05	3.6
SKBR3	72	45	0.13	0.11	3.7

Variance (**Variance**), standard deviation (**StDev**) and dynamic range (**DR**) of the hybridization of SKBR3 under the conditions of different hybridization duration (**Duration**) and temperature (**Temp.**).

**Table 3:** Hybridization temperature and dextran sulphate concentration

**Sample**	**Duration**	**Temp.**	**DS conc.**	**DS Mw.**	**Variance**	**StDev**	**DR**
SKBR3	72 h	37°C	10%	50 kDa	0.11	0.06	3.5
SKBR3	72 h	37°C	15%	50 kDa	0.11	0.07	3.7
SKBR3	72 h	42°C	10%	50 kDa	0.14	0.10	3.8
SKBR3	72 h	42°C	15%	50 kDa	0.12	0.09	3.8

Variance (**Variance**), standard deviation (**StDev**) and dynamic range (**DR**) for testing the effects of different dextran sulphate concentrations (**DS conc.**) and temperature (**Temp.**) at a hybridization duration (**Duration**) of 72 hours, hybridizing SKBR3.

**Table 4:** Dextran sulphate concentration

**Sample**	**Duration**	**Temp.**	**DS conc.**	**DS Mw.**	**Variance**	**StDev**	**DR**
SKBR3	72 h	37°C	15%	5 kDa	0.10	0.04	3.2
SKBR3	72 h	37°C	17.5%	5 kDa	0.10	0.05	3.7
SKBR3	72 h	37°C	20%	5 kDa	0.10	0.05	3.4
SKBR3	72 h	37°C	15%	10 kDa	0.11	0.04	3.7
SKBR3	72 h	37°C	17.5%	10 kDa	0.11	0.04	3.6
SKBR3	72 h	37°C	20%	10 kDa	0.11	0.03	3.6
SKBR3	72 h	37°C	15%	50 kDa	0.11	0.04	3.9
SKBR3	72 h	37°C	17.5%	50 kDa	0.11	0.04	3.7
SKBR3	72 h	37°C	20%	50 kDa	0.09	0.03	3.4

Variance (**Variance**), standard deviation (**StDev**) and dynamic range (**DR**) for testing the effects of different dextran sulphate concentrations (**DS conc.**) and molecular weights (**DS Mw.**), and temperature (**Temp.**) at a hybridization duration (**Duration**) of 72 hours, hybridizing SKBR3.

**Table 5:** pH of the hybridization buffer

**Sample**	**Hyb. pH**	**Variance**	**StDev**	**DR**	**Max CGHseg**	**Correlation**
FFPE tumor 2	6	0.07	0.04	1.4	0.6	0.98
FFPE tumor 2	6	0.08	0.04	1.6	0.7	

FFPE tumor 2	7	0.09	0.04	1.5	0.7	0.97
FFPE tumor 2	7	0.08	0.05	1.2	0.6	

FFPE tumor 2	8	0.08	0.04	1.3	0.6	0.94
FFPE tumor 2	8	0.10	0.05	1.7	0.8	

Variance (**Variance**), standard deviation (**StDev**), dynamic range (**DR**), the highest log2 ratio calculated by CGH-segmentation (**Max CGHseg**), and Pearson Correlation (**Correlation**) for testing the effects of pH (**Hyb. pH**) of the hybridization buffer on a CGH profile of FFPE tumor #2. The log2 ratios associated with homozygous deletions vary widely since they depend heavily on dividing very small intensities by large intensities. This can have disproportionate impact on the dynamic ranges, therefore only the highest ratio is taken as a measurement for the dynamic range (**Max CGHseg**).

**Table 6:** Wash temperature

**Sample**	**Wash**	**Variance**	**StDev**	**DR**	**Max CGHseg**	**Correlation**
FFPE tumor 2	65°C	0.09	0.04	1.5	0.7	0.97
FFPE tumor 2	65°C	0.08	0.05	1.2	0.6	

FFPE tumor 2	68°C	0.09	0.03	1.6	0.7	0.99
FFPE tumor 2	68°C	0.09	0.03	1.7	0.7	

Variance (**Variance**), standard deviation (**StDev**), dynamic range (**DR**), the highest log2 ratio calculated by CGH-segmentation (**Max CGHseg**), and Pearson correlation (**Correlation**) of FFPE tumor #2 washed after hybridization at 65°C and 68°C (**Wash**) in replicate.

**Table 7:** Manual and automated hybridization of FFPE tumors

**Sample**	**Hyb method**	**Variance**	**StDev**	**DR**	**Correlation**
FFPE tumor 3	manual	0.11	0.06	2.0	0.82
FFPE tumor 3	automated	0.10	0.04	1.9	

FFPE tumor 4	manual	0.10	0.05	1.4	0.72
FFPE tumor 4	automated	0.08	0.03	1.3	

FFPE tumor 5	manual	0.10	0.04	1.5	0.85
FFPE tumor 5	automated	0.08	0.02	1.3	

FFPE tumor 6	manual	0.13	0.06	1.9	0.84
FFPE tumor 6	automated	0.08	0.04	1.9	

Variance (**Variance**), standard deviation (**StDev**), dynamic range (**DR**) and Pearson correlation (**Correlation**) of four FFPE tumors hybridized with our manual and our automated method (**Hyb method**).
