# Supplementary figures and images for: Automated array-CGH optimized for archival formalin-fixed, paraffin-embedded tumor material
Source: BMC Cancer. 2007 Mar 7;7:43. doi: 10.1186/1471-2407-7-43 (PMC1829401; doi:10.1186/1471-2407-7-43)

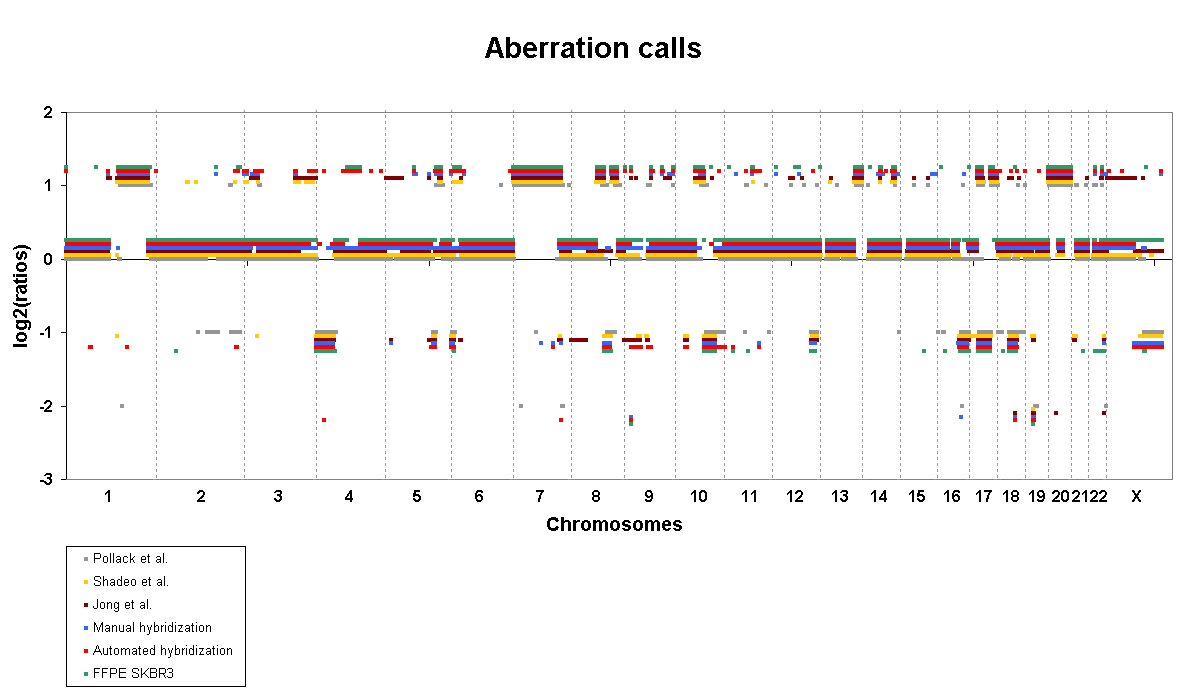

Supplement: Additional file 2 — This picture shows the copy number calls (Y-axis) from SKBR3 CGH profiles per chromosome (X-axis), hybridized by Pollack et al., Shadeo and Lam, Jong et al., manually, automatically, and hybridized FFPE SKBR3. Gain at 1, unchanged at 0, heterozygous loss at -1, and homozygous loss at -2. [file 1471-2407-7-43-S2.jpeg]
